# MiR-205-5p and MiR-222-3p as Potential Biomarkers of Endometrial Cancer

**DOI:** 10.3390/ijms26062615

**Published:** 2025-03-14

**Authors:** Anna Bogaczyk, Natalia Potocka, Sylwia Paszek, Marzena Skrzypa, Alina Zuchowska, Michał Kośny, Marta Kluz-Barłowska, Andrzej Wróbel, Jan Wróbel, Izabela Zawlik, Tomasz Kluz

**Affiliations:** 1 Department of Gynecology, Gynecology Oncology and Obstetrics, Fryderyk Chopin University Hospital, 35-055 Rzeszow, Poland; annabogaczyk@interia.pl (A.B.); jtkluz@interia.pl (T.K.); 2 Laboratory of Molecular Biology, Centre for Innovative Research in Medical and Natural Sciences, Faculty of Medicine, Collegium Medicum, University of Rzeszow, 35-959 Rzeszow, Poland; npotocka@ur.edu.pl (N.P.); mskrzypa@ur.edu.pl (M.S.); 3 Faculty of Medicine, Collegium Medicum, University of Rzeszow, 35-959 Rzeszow, Poland; spaszek@ur.edu.pl (S.P.); aowsiak@ur.edu.pl (A.Z.); 4 Department of Hematology, Medical University of Lodz, 90-419 Lodz, Poland; mkosny1@gmail.com; 5 Department of Pathology, Fryderyk Chopin University Hospital, 35-055 Rzeszow, Poland; marta.kluz@interia.pl; 6 Second Department of Gynecology, Medical University of Lublin, Jaczewskiego 8, 20-090 Lublin, Poland; wrobelandrzej@yahoo.com; 7 Medical Faculty, Medical University of Lublin, 20-090 Lublin, Poland; wrobeljan@onet.eu

**Keywords:** microRNA, miR-21-5p, miR-205-5p, miR-222-3p, endometrium cancer, EC, dPCR, digitalPCR, biomarkers

## Abstract

Endometrial cancer is the fourth most common cancer in women in Europe. Its carcinogenesis is a complex process and requires further research. In our study, we focus on finding new and easy-to-diagnose markers for detecting endometrial cancer. For this purpose, we compared the levels of miR-21-5p, miR-205-5p, and miR-222-3p in endometrial cancer tissues with the levels of these miRs in the serum of patients using the dPCR method. Our study is preliminary and consists of comparing the changes in miRNA expression in serum to the changes in miRNA in tissue of patients with endometrial cancer. The study included 18 patients with EC and 19 patients undergoing surgery for pelvic organ prolapse or uterine fibroids as a control group without neoplastic lesions. Endometrial tissue and serum were collected from all patients. The analyses showed an increased expression of miR-205-5p in endometrial cancer tissue and decreased expression of miR-222-3p in tissue and serum samples. These results suggest that miR-205-5p and miR-222-3p may be potential endometrial cancer biomarkers. Only miR-222-3p confirmed its decreased expression in serum, making it a potential and easily accessible marker in the diagnosis of endometrial cancer. This pilot study requires further investigation in a larger group of patients. Its advantages include the possibility of a comparison between miRNA expression in tissue and serum, as well as conducting the study using dPCR.

## 1. Introduction

Endometrial cancer is the fourth most common female cancer in Europe. Its incidence is 12.9–20.2 per 100,000 women and its mortality is 2.0–3.7 per 100,000 women [1,2]. The most commonly used divisions of EC are morphological and molecular profiling. In the morphological division, according to Bokhman, EC is divided into type I, endometrioid carcinoma, which is associated with excess estrogens. It develops as a result of endometrial hyperplasia. This type occurs more often and has a good prognosis. The second type, non-endometrioid, is not associated with estrogen stimulation and has a poor prognosis [3]. A newer division is molecular profiling, which was introduced in 2013 by The Cancer Genome Atlas (TCGA). According to this division, EC is divided into four molecular subgroups:

– POLEmut group, which is characterized by POLE mutation, and accounts for 7%;
– Microsatellite instability (MSI group), resulting from MMR-deficient repair deficiency. It accounts for 28% and has a relatively favourable prognosis. The most common mutations in the MSI group include mutations in the ARID5B, PTEN, and phosphatidylinositol-3 kinase families, including PIK3CA and PIK3R1;
– High somatic copy number changes (serous group, driven by a TP53 mutation, also called the p53abn group), constitute 26%;
– A low copy number group without a specific driver mutation (NSMP group), constitutes 39% [4].

POLE-mutated tumours have a good prognosis, while the high-copy group driven by the TP53 mutation has a poor prognosis. The prognosis of mismatch repair-deficient tumours and those without a defined molecular profile (NSMP) is relatively favourable [4,5,6]. Molecular staging of endometrial cancer is useful because of its prognostic value and potential to predict the benefits of adjuvant therapy [2].

The stage of endometrial cancer has been assessed so far in surgical and pathological stages based on the FIGO classification from 2009 (Table 1) [7]. Due to the introduction of the new molecular classification, the ESGO/ESTRO/ESP guidelines were developed, which added new subclassifications to the previous grading system, and introduced the new FIGO 2023 classification (Table 2) [8].

Despite the knowledge on various genetic mutations in endometrial cancer (microsatellite instability and mutations in PTEN, K-ras, beta-catenin, p53, HER-2/neu, p16 and E-cadherin genes) and distinguishing different genetic types of this cancer, we still lack knowledge of the regulatory mechanisms occurring in this cancer [9,10,11]. Therefore, in our study, we focus on miRNA and changes in its expression both in EC tissues and serum in patients with endometrial cancer.

MiRNAs are non-coding, single-stranded, small RNA molecules with a length of approximately 19–25 nucleotides. They were discovered in 1993 and described by Lee et al. as the first to describe small RNA molecules encoded by the gene regulating the expression of the lin-14 protein in Caenorhabditis elegans [12].

MiRNAs are formed from primary miRNAs (pri-miRNAs) in the cell nucleus with the participation of RNA polymerase II, the Drosha protein complex and DGCR8. Subsequently, in the cytoplasm, precursor miRNAs (pre-miRNAs) are cleaved by Dicer to generate mature miRNAs [13]. MiRNAs are remarkably stable in various body fluids, including saliva [14], urine [15], breast milk [16,17], and blood [18]. To maintain stability outside the cell, they must be protected against degradation and the action of endonucleases. MiRNAs are packaged into exosomes or microvesicles. Alternatively, they can be attached to high-density lipoproteins (HDL) or bound by the AGO2 protein outside the vesicles. These protective mechanisms ensure the stability of extracellular miRNAs [19,20,21].

MiRNAs have been extensively studied as potential biomarkers in the serum of patients with endometrial cancer. Below, some selected examples have been discussed.

Tan et al. reported that hsa-miR-155 might be a good diagnostic marker. Its serum level was increased in patients with endometrial cancer and increased with the stage of EC, as well as with lymph node metastasis [22].

Several miRNAs, such as miR-15a-5p, miR-106b-5p, and miR-107, were significantly upregulated in exosomes isolated from the plasma of EC patients compared to healthy individuals. Notably, plasma-derived miR-15a-5p appears to be a promising and effective diagnostic biomarker for the early detection of endometrial cancer, particularly in differentiating stage I patients from healthy controls [23].

Additionally, serum levels of miR-204-5p were found to be decreased in EC patients compared to those with benign lesions. This reduction was particularly pronounced in patients with lymph node metastases, highlighting its potential as an early diagnostic biomarker [18].

MiR-27a-5p, another miRNA of interest, was significantly increased in serum exosomes of patients with polycystic ovary syndrome (PCOS), and may play a role in the development of EC in this patient population [24].

Similarly, miR-887-5p expression was elevated in the serum of EC patients compared to healthy individuals, indicating its potential diagnostic value [25].

Kumari et al. observed the dysregulated expression of several miRNAs in the serum of EC patients: miR-16, miR-99b, miR-125, and miR-145 were downregulated, while miR-143 was upregulated [26].

In another study, four miRNAs, miR-222, miR-223, miR-186, and miR-204, were significantly increased in the serum of EC patients compared to controls, further supporting their potential as biomarkers for this cancer [27].

Ghazala et al. studied miR-27a and miR-150-5p in the serum of EC patients and both miRNAs may be promising EC biomarkers [28].

Also, miR-203 expression levels were higher in the serum of EC patients, but its levels were not correlated with promoter methylation status [29].

The miRNAs used in our study (miR-21-5p, miR-205-5p and miR-222-3p) have been studied in various other cancers and are involved in carcinogenesis, which is why they were selected for testing in EC.

MiR-21-5p has been extensively studied in various cancers, for example, in colorectal cancer where it showed strong expression in serum samples from patients, while its levels were significantly downregulated in postoperative patients. Its high expression was correlated with TNM testing and lymph node metastasis [30]. MiR-21-5p has also been utilized as a biomarker for the detection and prognosis of pancreatic cancer [31], and non-small cell lung cancer (NSCLC) [32]. High levels of miR-21-5p in serum were also observed in oropharyngeal cancer associated with Epstein–Barr virus infection [33].

In breast cancer, however, miR-21-5p showed reduced expression and limited diagnostic value for early-stage disease. Nonetheless, its reduced expression may serve as a biomarker in the diagnosis of breast cancer metastases [34].

MiR-21 is one of the most consistently expressed miRNAs in almost all types of human cancer [35]. Its expression level is also altered in EC cells, as shown by Sato et al. They assessed miR-21 expression in EC tumour cells and stroma separately. Upregulation of miR-21 in EC cells was significantly associated with higher histological grade and lymph node metastasis. It was also significantly associated with poor progression-free survival [36]. Bouziyane et al. also confirmed the diagnostic value of miR-21 in EC tissue. The investigators suggest that tests with blood, serum, or urine should be performed [37].

The next miR under investigation was miR-205-5p, where its levels in serum were significantly higher in patients with non-small cell lung cancer compared to controls [38]. Jiang et al. observed similar changes in miR-205-5p levels in non-small cell lung cancer [39]. MiR-205-5p was also tested in serum to differentiate benign from malignant thyroid tumours. Serum levels were the highest in patients with malignant thyroid tumours, followed by those with benign tumours, and were the lowest in the control group. Additionally, its expression correlated with tumour size, stage, lymph node metastases, tumour capsule invasion, and the *BRAF* mutation status were reported. A positive correlation between miR-205-5p expression and TSHR mRNA levels was identified in thyroid cancer patients [40].

MiR-205 expression was also examined in EC tissue. Karaayvaz et al. confirmed increased miR-205 expression in EC cells. The increased expression was not associated with disease stage or tumour type. The investigators found that higher miR-205 expression was significantly associated with overall shorter patient survival [41]. Jin et al. also showed that miR-205 played an important role in the migration and invasion of endometrial cancer [42]. Other researchers also confirmed the oncogenic effect of miR-205 on EC cells. However, in all cases the material studied was endometrial cancer tissue [43,44]. MiR-205 expression also plays a role in the treatment of endometrial cancer. The researchers showed that silencing of miR-205-5p increased the sensitivity of EC cells to paclitaxel (PTX), which resulted in reduced cell proliferation and increased apoptosis. In contrast, increased expression of miR-205-5p increased PTX resistance and contributed to EC cell tumourigenesis [45].

The third miR that took part in the study was miR-222-3p. It was detected in serum exosomes from ovarian cancer patients, and its levels were associated with epithelial ovarian cancer (EOC). Exosomal miR-222-3p is an effective regulator of tumour-promoting M2 macrophage polarization [46].

In another study, exosomal miR-222-3p was tested in patients with papillary thyroid cancer and was significantly increased in patients with lymph node metastasis [47].

This miR has also been studied in EC. Liu et al. studied miR-222 in women with endometrial cancer and confirmed its effect on tumourigenesis. Only EC tissue was used in this study [48].

Furthermore, serum expression levels of four miRNAs, including miR-21-5p and miR-222-3p, were investigated in NSCLC patients and demonstrated good diagnostic value, particularly in early-stage disease [49].

Many studies have been conducted to identify various miRNAs associated with EC cells. However, further understanding of the factors regulating carcinogenesis is needed to identify markers that facilitate their detection and to discover targets that could serve as therapeutic targets.

In this study, we focused on comparing the levels of three miRNAs (miR-21-5p, miR-205-5p, and miR-222-3p) in endometrial cancer tissues with the levels of the same miRNAs in the serum of patients using dPCR. We believe that the use of serum testing in potential endometrial cancer patients is a better method than tissue testing, due to the ease of obtaining the material for testing and a lower risk of complications.

## 2. Results

The clinical characteristics and lifestyle data of patients with endometrial cancer are presented in Table 3. Due to the relatively small size of the control group and the presence of non-normal data distributions, the Mann–Whitney U test was used to analyze quantitative variables. No statistical significance was obtained for the following factors: age of first and last menstrual period, births, BMI, and hypertension. However, statistically significant differences were identified for patient age, diabetes mellitus (DM), and hypothyroidism. Table 4 presents the patients’ clinicopathological data, including numerical and percentage distribution depending on the stage (FIGO), and differentiation.

The absolute expression of miR-21-5p, miR-205-5p, and miR-222-3p was measured in endometrial cancer tissue and compared with absolute expression research miRNAs in the control group. Statistically significant differences were observed for miR-205-5p (*p* < 0.001) and miR-222-3p (*p* = 0.003), as shown in Table 5. Reference genes U6 and SNORD48 were included in the study but were found to be unstable, and, therefore, not included in the analyses, as described in our 2024 paper [50].

The data were further processed using logarithmic transformation to normalize the distribution. Mean logarithmic values were used to calculate fold change (FC). In addition, both univariate and multivariate random regression analyses were performed.

Table 6 presents the expression levels of microRNAs in the studied endometrial cancer tissue and control group as logarithmic data. The statistical analyses indicated that, amongst the three microRNAs that were examined, microRNA-205-5p exhibited significant upregulation (*p* < 0.001), whereas microRNA-222-3p demonstrated significant downregulation (*p* < 0.001). Following the application of the Benjamini–Hochberg correction, the disparities in the concentration of miRNAs between the study and control groups for miR-205-5 persisted at a significance level of *p* < 0.001, while for miR-222-3p, the adjusted *p*-value was 0.002. However, the expression of miR-21-5p remained non-significant.

Subsequently, the absolute levels of miR-21-5p, miR-205-5p, and miR-222-3p in serum were analyzed in the aforementioned group of patients with endometrial cancer and compared to their absolute expression in the control group. A statistically significant difference was observed for miR-222-3p expression (*p* = 0.019). The external control, cel-miR-39-3p, exhibited comparable levels in both groups. Table 7 presents the absolute serum expression of the analyzed miRNAs in copies per µL.

The raw data obtained from the dPCR for serum have been processed in the same way as the data obtained for tissue. Univariate and multivariate random regression analyses were carried out, incorporating logarithmic transformation to normalize the data distribution. The expression levels of microRNAs in the serum of patients with endometrial cancer and the control group, presented as logarithmic values, are shown in Table 8. Among the three analyzed miRNAs, only miR-222-3p exhibited statistically significant downregulation (*p* = 0.011). After implementing a Benjamini–Hochberg correction, an adjusted *p*-value of 0.022 was obtained, thus confirming the observed difference between the study and control groups. In contrast, no statistically significant differences were found for miR-21-5p and miR-205-5p.

We performed a univariate logistic regression analysis of clinical variables and miRNA expression levels to investigate potential factors associated with the occurrence of EC.

Age is regarded as an important clinical factor associated with the development of EC (OR 1.09, 95%CI: 1.01–1.17, *p* = 0.029). BMI showed borderline significance. Among the tested miRNAs, two showed significant associations with endometrial cancer in tissue: miR-205-5p (OR 10.91, 95%CI: 2.27–52.48 *p* = 0.003) and miR-222-3p (OR 0.02, 95%CI: 0.002–0.35, *p* = 0.006). In serum, statistical significance was obtained for miR-222-3p (OR 0.12, 95%CI: 0.02–0.74, *p* = 0.022) (Table 9).

**Figure 1:**
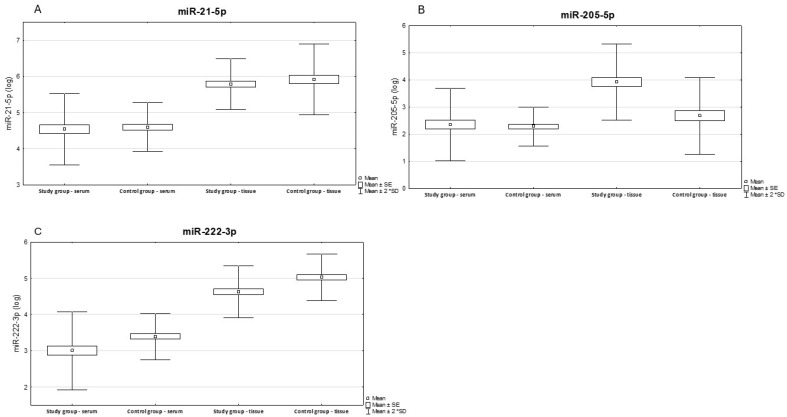
Illustrates the differences in expression levels between tissue and serum in the study and control groups. Results of paired serum-tissue *t*-tests for the control and study groups. Results expression miR-21-5p (**A**), miR-205-5p (**B**), miR-222-3p (**C**).

We have presented below the results of paired *t*-test analysis for tissue and serum in the study and control groups. Figure 1 shows logarithmic data illustrating the differences in expression levels of the tested miRNAs between tissue and serum in the study group compared to the control group.

The correlation between miRNA concentration in serum and tissue was established using Spearman’s correlation test, but statistical significance was not obtained (Table 10).

We also performed multivariate logistic regression for the occurrence of endometrial cancer, but the results were not statistically significant (Table 11).

Regarding the stage of advancement, 16% of patients had FIGO => 2. We performed a complete set of logistic regressions for FIGO < 2, or => 2—none of the results reached statistical significance. Therefore, we evaluated the differences in concentrations in the given groups, in serum miR-205-5p was initially statistically significant (*p* = 0.017), but lost significance with the Benjamini–Hochberg correction for multiple comparisons (*p* = 0.17) (Table 12).

Among patients with endometrial cancer, 33% were patients with the G3 differentiation stage. We evaluated the differences in concentrations in the G3 and non-G3 groups.

No statistically significant differences in miRNA expression between the groups were observed in tissue. However, in serum, the concentration of miR-205-5p was initially significant (*p* = 0.024) but lost significance after applying the Benjamini–Hochberg correction (*p* = 0.12) (Table 13).

## 3. Discussion

Our study is preliminary as it is conducted on a small group of patients and requires the study group to be expanded. The major advantage is the use of dPCR measurements, and the application of this method distinguishes our study from other similar studies cited below. An important aspect of our study is that we used free-circulating microRNA rather than the isolated one from exosomes. Serum is a material that can be collected from the patient in a minimally invasive manner, and the direct isolation of microRNA is easy to perform in routine studies. However, the difficulty is that we cannot directly compare the results of free-circulating miRNA expression with those studies where the isolated miRNA was from exosomes.

When examining endometrial cancer tissue, we obtained statistically significant results for absolute miR-205-5p expression (*p* < 0.001), which did not change after Benjamini–Hochberg correction.

These findings align with previous studies, such as Karaayvaz et al., who highlighted the prognostic potential of miR-205 in endometrial cancer and demonstrated its upregulation [41]. Lu et al. and others also reported similar findings, underscoring the oncogenic role of this miRNA in endometrial cancer. Lu et al. also detected upregulation of miR-205-5p [42,43,44,45]. However, in our study, miR-205-5p expression was not statistically significant in serum. This discrepancy may result in differences in miRNA stability or regulatory mechanisms between tissue and serum. In contrast to our results, its established role had been previously described in other cancers, such as ovarian cancer, non-small cell lung cancer or breast cancer [38,51,52]. The researchers investigated the changes in miR-205 expression in serum of patients with non-small cell lung cancer, and in serum of patients with breast cancer, only in the case of ovarian cancer, exosome-derived miRNAs were used in the studies. The role of miR-205 was also described in animal serum [53].

Using univariate logistic regression, we confirmed the significant role of miR-205-5p as an endometrial cancer factor, also only in the EC tissue.

After dividing the patients according to the stage of advancement (FIGO < 2 and FIGO => 2) we assessed the differences in concentrations in the individual groups and miR-205-5p was initially statistically significant (*p* = 0.017), but lost statistical significance after applying the Benjamini–Hochberg correction (*p* = 0.17).

A similar situation occurred after dividing the patients according to the degree of differentiation (G3 and non-G3) we assessed the differences in concentrations in the individual groups and miR-205-5p was initially statistically significant (*p* = 0.024), but lost statistical significance after applying the Benjamini–Hochberg correction (*p* = 0.12). Our results are promising and indicate that studies in larger groups of patients are required to confirm the clinical usefulness of miR-205-5p in these subgroups.

The next miR examined was miR-222-3p, which was statistically significant in the tissue (*p* < 0.001), but after the Benjamini–Hochberg method was applied, it was corrected to 0.002. This miR was studied by Liu et al. and confirmed its effect on tumourigenesis in endometrial cancer tissues. The researchers, examining EC tissue, found that miR-222-3p expression correlated with ERα. The expression level of miR-222-3p was the lower in lower stage (1 and 2 vs. 3) and earlier stage (I vs. II, II vs. III, I vs. III) tumours. Furthermore, miR-222-3p was positively associated with lymph node metastasis [48].

In other cancers, researchers have also reported its oncogenic role after studying cancer tissue, for example, in osteosarcoma or lung cancer [54,55]. Exosomal miR-222 can also be studied in serum, e.g., its oncogenic effect on ovarian cancer [46]. The suppressive role is described by Fu et al. in their study on ovarian cancer [56]. In our study, we detected the downregulation of miR-222-3p expression in both tissue and serum of EC patients, indicating its suppressive role in EC cells. When examining serum in the group of patients with endometrial cancer, only miR-222-3p remained statistically significant (*p* = 0.011). However, our study showed no correlation between serum and tissue miR data (Table 7).

In contrast, miR-21-5p did not show significant changes in either tissue or serum in our study, despite previous reports linking its high expression to faster disease progression, lymph node metastases, and high diagnostic efficacy in EC [36,37]. This discrepancy underscores the need for further research to clarify its role and diagnostic value.

The observed discrepancies between tissue and serum miRNA expression patterns, as well as between our results and prior studies, highlight the complexity of miRNA regulation in endometrial cancer. While our findings provide valuable insights, they also underscore several limitations, including the need for larger sample sizes to confirm statistically robust results and more comprehensive analyses to explore the mechanistic roles of miRNAs and reference genes in EC.

## 4. Materials and Methods

### 4.1. Tissues and Serum Samples

The study involved 18 patients diagnosed with endometrial cancer. All diagnoses were confirmed by previous histopathological tests. These patients came to the Department of Gynecology, Gynecology Oncology and Obstetrics of the Fryderyk Chopin University Hospital in Rzeszów between 04/2021 and 11/2022 to start oncological treatment. In our study, endometrial cancer tissue material, as well as serum from patients with endometrial cancer were used. The patients constituted the study group. The study also included 19 healthy women (without endometrial cancer) operated on in the local clinic due to pelvic organ prolapse or uterine fibroids, whose serum was collected as a control and these patients constituted the control group. All women consented to the use of tissues for genetic testing. The local bioethics committee approved the research project. Consent of the Bioethics Committee of the District Medical Chamber of 21 May 2020, resolution No. 54/B/2020. None of the patients received hormonal therapy, radiotherapy, or chemotherapy before sample collection.

### 4.2. miRNA Isolation from Serum Samples

Blood collected from the patients was centrifuged twice and the resulting serum was stored at −80 °C. Serum samples from endometrial cancer were thawed on ice. Isolation of total RNA, including miRNA, from serum was performed using the miRNeasy Serum/Plasma Advanced Kit (Cat. No. 217204, Qiagen, Hilden, Germany) according to the manufacturer’s protocol. MiRNA isolation was performed from 400 µL serum, adjusting buffer volumes for larger starting sample volumes. To establish internal standards for subsequent steps, 1 µL of spike-in control was added to the samples. After centrifugation, the upper aqueous phase containing total RNA, including miRNA, was transferred to a new reaction tube. Isopropanol was added to the supernatants to provide the appropriate conditions for RNA molecules (>18 nucleotides) to bind to the silica membrane. The entire sample was transferred to the RNeasy UCP MinElute centrifuge column, where subsequent wash steps removed membrane-bound contaminants. For RNA elution, 20 µL of RNase-free water was used. The microRNA from samples were frozen at −20 °C.

### 4.3. miRNA Isolation from Tissue Samples

Tissue samples from patients with endometrial cancer were collected and preserved in RNAprotect Tissue Reagent (Cat. No. 76104, Qiagen, Hilden, Germany) to maintain RNA integrity. These samples were then frozen at −80 °C. Total RNA, including miRNA, was extracted using the miRNeasy Mini Kit (Cat. No. 217084, Qiagen, Hilden, Germany) per the manufacturer’s instructions. The frozen tissues of approximately 10-20 mg were initially thawed on ice and transferred into tubes containing 700 µL of QIAzol Lysis Reagent. The samples were homogenized using sonication, followed by the addition of chloroform. The mixture was centrifuged at 12,000× *g* for 15 min at 4 °C to separate the phases. The upper aqueous phase, which contains total RNA including miRNA, was carefully transferred to new tubes, mixed with ethanol, and then loaded onto RNeasy MiniElute Spin Columns for further purification. To remove any contaminating DNA, the samples underwent on-column digestion using the RNase-free DNase kit (Cat. No. 79254, Qiagen, Hilden, Germany). RNA was eluted in 30 µL of RNase-free water. The RNA concentration and purity were determined using the NanoDrop™ 2000c Spectrophotometer (ThermoFisher Scientific, Waltham, MA, USA), and the samples were diluted to a final RNA concentration of 5 ng/µL. To assess RNA integrity, electrophoresis was performed on a 1% agarose gel. Reverse transcription of the isolated RNA was carried out immediately following the isolation procedure to ensure RNA stability and quality.

### 4.4. Reverse Transcriptase Reaction

MicroRNAs were subjected to polyadenylation through the use of a poly(A) polymerase, and subsequently reverse-transcribed into cDNA using oligo-dT primers with a degenerate 3′ anchor sequence, thereby facilitating the amplification of miRNAs during a real-time PCR reaction. Polyadenylation and reverse transcription were conducted in parallel in the same tube. cDNA synthesis was performed according to the manufacturer’s instructions using the miRCURY LNA Reverse Transcription Kit (Cat. No. 339340, Qiagen, Hilden, Germany). The final volume of the RT reaction mixture was 10 µL and consisted of 2 µL RNA, 2 µL of 5× miRCURY SYBR^®^ Green RT Reaction Buffer, 1 µL of 10× miRCURY RT Enzyme Mix, 0.5 µL of both UniSp6 and miR-cel-39-3p spike-ins and 4.5 µL of RNase-free water. The RT reaction was carried out in a T100™ 96-well thermocycler (Bio-Rad, Hercules, CA, USA) under the following conditions: incubation at 42 °C for 60 min, inactivation at 95 °C for 5 min, then the whole reaction was cooled to 4 °C. The cDNA was stored frozen at −20 °C until further use.

### 4.5. Absolute Quantification by dPCR

Absolute quantification for three miRNAs: hsa-miR-21-5p, hsa-miR-205-5p, and hsa-miR-222-3p was determined in serum samples from all patients in the study and control groups using QIAcuity System dPCR (Qiagen, Hilden, Germany). Cellular miR-39 was selected as the exogenous reference gene only in serum. The dPCR technique employs microfluidic nanoplate technology. This approach enables the quantification of nucleic acids by measuring the fluorescence endpoint of each partition. To determine the absolute concentration, we used the miRCURY LNA miRNA PCR Assay (Cat. No. 339306, respectively: miR-21-5p YP00204230; miR-205-5p YP00204487; miR-222-3p YP00204551; cel-miR-39-3p YP00203952; Qiagen, Hilden, Germany) using QIAcuity Nanoplate (Qiagen, Hilden, Germany) 26K 24-well plates for miRNAs isolated from serum (Cat. No. 250001), and an 8.5K 96-well plate for miRNAs isolated from tissue (Cat. No. 250021). The dPCR reaction was performed using the QIAcuity EG PCR Kit (Cat. No. 250112, Qiagen, Hilden, Germany) as recommended by the manufacturer. Each dPCR mixture consisted of 13.3 µL Eva Green PCR Master Mix, 4 µL primer mix, 10 µL of cDNA template (diluted 1:20 for cDNA derived from miRNA), and 12.7 µL of RNase-free water. A total of 40 µL of the reaction mixture was added to each well of a 24-well Nanoplate 26K and amplified under the following conditions: heat activation at 95 °C for 2 min, followed by 40 amplification cycles of denaturation at 95 °C for 15 s, annealing at 60 °C for 15 s and extension 72 °C for 15 s. The fluorescence was measured after all the cycles. Expression levels for the miRNAs tested were calculated based on the concentration expressed by the number of miRNA copies in the sample. The calculations were carried out using QIAcuity Software Suite version 2.1.8 (Qiagen, Hilden, Germany).

### 4.6. Statistical Analysis

The expression of miRNAs quantified with dPCR was log-transformed in order to achieve a normal distribution of data. Next, miRNA stability analysis was assessed using NormiRazor. NormiRazor is a tool that implements three different existing normalization algorithms—geNorm, NormFinder and BestKeeper [57]. None of the miRNAs were found to be stable enough to be considered acceptable normalizers. Differential expression analysis was conducted using an independent *t*-test, Welch *t*-test, or U Mann–Whitney test, selected based on variable distribution and variance equality, as determined by the Shapiro–Wilk test and Levene’s test, respectively. To control the false discovery rate associated with multiple comparisons, the Benjamini–Hochberg method was applied. Paired *t*-test was used to evaluate differences between miRNA concentration in serum and tissue. Spearman’s rank correlation test was used to assess correlation between serum and tissue concentration of miRNA. Univariate logistic regression analysis was applied to evaluate the impact of specific factors on the occurrence of endometrial cancer. Variables found to be significant in the univariate analysis were subsequently included in a multivariate logistic regression model. Nominal variables are reported as counts and percentages or as median (with range or interquartile range) and mean (±SD), depending on the normality of their distribution. Statistical analyses were performed using Statistica 13.1 software (TIBCO, Palo Alto, CA, USA) and R version 4.2.1. A *p*-value of less than 0.05 was considered statistically significant.

## 5. Conclusions

The analyses demonstrated increased expression of miR-205-5p in endometrial cancer tissue and decreased expression of miR-222-3p in both tissue and serum samples. These findings suggest that miR-205-5p and miR-222-3p may serve as potential biomarkers for endometrial cancer. Further studies on a larger group are needed to elucidate the diagnostic utility of microRNAs (miRNAs) such as miR-205 and miR-222.

## 6. Limitations

Our study had some limitations. The first was the small group of patients participating in the study, as the study group consisted of 18 patients with EC and the control group consisted of 19 patients. The obtained results of changes in miRNA expression are very promising, but our study only evaluates miRNAs and further studies with other miRNAs involved in EC carcinogenesis are necessary to better understand this process.

## Figures and Tables

**Table 1:** FIGO 2009 classification of endometrial cancer.

	FIGO 2009
Stage I	Confirmed to the corpus uteri
IA	No or less than half myometrial invasion
IB	Invasion equal to more than half of the myometrial
IC	
Stage II	Invasion of cervical stroma without extrauterine extension
IIA	
IIB	
IIC	
Stage III	Local and/or regional spread of the tumour
IIIA	Tumour invades the serosa of the corpus uteri and/or adnexa
IIIB	Vaginal involvement and/or parametrial involvement
IIIC	Metastases to pelvic and/or para-aortic lymph nodes
	IIIC1 Positive pelvic nodes
	IIIC2 Positive paraaortic nodes with or without positive pelvic lymph nodes
Stage IV	Tumour invades bladder and/or bowel mucosa, and/or distant metastases

**Table 2:** New FIGO 2023 classification of endometrial cancer.

	FIGO 2023
Stage I	Limited to the uterine body and ovary
IA	Tumour occurring only in the endometrium OR non-aggressive histological type (low-grade EC, with invasion of less than half of the uterine muscle, with negative LVSI or with focal LVSI involvement OR tumour with good prognosis
	IA1 Non-aggressive histological type occurring in a polyp in the endometrium OR confined to the uterine mucosa
	IA2 Non-aggressive histological types involving less than half of the myometrium with negative or positive focalLVSI
	IA3 Low-grade EC limited to the uterus and ovaries
IB	Non-aggressive types of EC with invasion of half or most of the myometrium and with or without focal LVSI
IC	Aggressive histological types occurring in polyps or confined to the uterine mucosa
Stage II	Invasion of cervical stroma without extrauterine extension OR with substantial LVSI OR aggressive histological types with myometrial invasion
IIA	Non-aggressive histological types of EC involving the stroma of the cervix
IIB	Non-aggressive histological types including substantial LVSI
IIC	Aggressive histological types with any myometrial involvement
Stage III	Local and/or regional EC infiltration of any histological subtype
IIIA	Invasion of uterine serosa, adnexa, or both by direct extension or metastasis
	IIIA1 Invasion of ovary or fallopian tube (outside of grade IA3 criteria)
	IIIA2 Invasion of uterine serosa or extension through uterine serosa
IIIB	Invaginal and/or parametrial or pelvic peritoneal EC
	IIIB1 Invaginal and/or parametrial EC
	IIIB2 Metastases to pelvic peritoneum
IIIC	Metastasis to the pelvic or para-aortic lymph nodes or both
	IIIC1 Metastasis to the pelvic lymph nodes IIIC1i Micrometastasis IIIC1ii Macrometastasis
	IIIC2 Metastases to para-aortic lymph nodes up to renal vessels, with or without metastases to pelvic lymph nodes IIIC2i Micrometastasis IIIC2ii Macrometastasis
Stage IV	Tumour invasion of the bladder mucosa and/or intestinal mucosa and/or distant metastases
	IVA Invasion of the bladder mucosa and/or the intestinal/bowel mucosa
	IVB Abdominal peritoneal metastasis beyond the pelvis
	IVC Distant metastasis, including metastasis to any extra- or intra-abdominal lymph nodes above the renal vessels, lungs, liver, brain, or bone

**Table 3:** Characteristics and clinical data of the study and control groups.

Characteristic	Overall, n = 37 ^1^	Study Group, n = 18 ^1^	Control Group, n = 19 ^1^	*p*-Value ^2^
Age	53 (42–92)	60 (48–82)	48 (42–92)	**0.002**
First period	14 (11–17)	13 (11–16)	14 (11–17)	0.2
Last menstrual period	50 (40–61)	50 (40–58)	48 (42–61)	0.2
Births	2 (0–4)	2 (0–4)	2 (0–3)	0.6
BMI	26.4 (20.08–50.2)	27.25 (22.1–50.2)	25.39 (20.08–34.19)	0.058
Hypertension				0.2
No	27 (73%)	11 (61%)	16 (84%)	
Yes	10 (27%)	7 (39%)	3 (16%)	
DM				**0.046**
No	33 (89%)	14 (78%)	19 (100%)	
Yes	4 (11%)	4 (22%)	0 (0%)	
Hypothyroidism				**0.020**
No	32 (86%)	13 (72%)	19 (100%)	
Yes	5 (14%)	5 (28%)	0 (0%)	

^1^ Median (Range); ^2^ U Mann–Whitney Test, Chi 2test with Fisher’s exact test depending on variable. Statistical significance was demonstrated in the following cases: patient’s age, diabetes (DM), and hypothyroidism. The *p*-values for statistically significant results are in bold.

**Table 4:** Clinicopathological data of the study group including patients with EC.

Degree of Differentiation:	Number of Patients Examined:	Percentage of Female Patients Surveyed
G1	0	0
G2	12	67%
G3	6	33%
FIGO stage:		
FIGO I	14	78%
FIGO II	2	11%
FIGO III	2	11%

**Table 5:** Absolute expression of miR-21-5p, miR-205-5p, and miR-222-3p in the tissue of the endometrial cancer study group and in the control group.

Absolute Expression (Copies/µL)	Study Group, N = 18 ^1^	Control Group, N = 19 ^1^	*p*-Value ^2^
miR-21-5p	655,024 (161,648–2,061,856)	938,736 (118,688–3,424,496)	0.3
miR-205-5p	10,164 (381.52–113,472)	181.52 (0.0–7336.8)	**<0.001**
miR-222-3p	50,132 (10,192–145,704)	112,168 (35,104–421,912)	**0.003**

^1^ Median (Range); ^2^ U Mann–Whitney Test. Statistically significant results were obtained for the expression of miR-205-5p (*p* < 0.001), and miR-222-3p (*p* = 0.003). The absolute expression of the tested miRNAs is presented in copies per µL. The *p*-values for statistically significant results are in bold.

**Table 6:** Expression of miRNAs in endometrial cancer tissue (N = 18).

miRNA	Study Group		Control Group		Fold Change	log_2_FC	*p*-Value ^1^	Benjamini–Hochberg Adjusted *p* Value ^2^
	Mean	SD	Mean	SD				
miR-21-5p	5.79	0.35	5.92	0.49	0.98	−0.03	0.36	0.576
miR-205-5p	3.93	0.7	2.68	0.71	1.47	0.55	**<0.001**	**<0.001**
miR-222-3p	4.63	0.36	5.03	0.32	0.92	−0.12	**<0.001**	**0.002**

^1^ The comparisons were performed using independent *t*-test and Mann–Whitney U-test depending on the distribution. The expression level of miRNAs in the endometrial cancer tissue studied was presented as logarithmic data. MiR-205-5p was shown to be upregulated, while miR-222-3p was downregulated; ^2^ The Benjamini–Hochberg correction was used to account for multiple comparisons. The *p*-values for statistically significant results are in bold.

**Table 7:** Absolute expression of miR-21-5p, miR-205-5p, and miR-222-3p in serum of the study group with endometrial cancer and the control group.

Absolute Expression (Copies/µL)	Study Group, N = 18 ^1^	Control Group, N = 19 ^1^	*p*-Value ^2^
cel-miR-39-3p	16,200 (2825.6–22,320)	17,664 (4682.4–49,608)	0.408
miR-21-5p	36,768 (4920.8–231,216)	38,832 (5545.6–157,976)	0.599
miR-205-5p	203.88 (17.9–3722)	260.8 (26.66–492.8)	0.799
miR-222-3p	964.2 (74.86–7570)	2366 (301–10,114)	**0.019**

^1^ Median (Range); ^2^ U Mann–Whitney Test. Statistically significant results were obtained for the expression of miR-222-3p (*p* = 0.019). The absolute expression of the tested miRNAs are presented in copies per µL. The *p*-values for statistically significant results are in bold.

**Table 8:** Serum miRNA expression in patients with endometrial cancer (N = 18).

miRNA	Study Group		Control Group		Fold Change	log_2_FC	*p*-Value ^1^	Benjamini–Hochberg Adjusted *p* Value ^2^
	Mean	SD	Mean	SD				
miR-39-3p	4.14	0.21	4.20	0.28	0.99	−0.02	0.403	0.576
miR-21-5p	4.54	0.50	4.60	0.34	0.99	−0.02	0.664	0.778
miR-205-5p	2.35	0.67	2.28	0.36	1.03	0.04	0.700	0.778
miR-222-3p	3.00	0.54	3.39	0.32	0.89	−0.18	**0.011**	**0.022**

^1^ Comparisons were performed using independent *t*-test and Mann–Whitney U test depending on the distribution. The expression level of miRNAs in the tested endometrial cancer serum is presented as logarithmic data. MiR-222-3p was shown to be downregulated, ^2^ Benjamini–Hochberg correction was used to account for multiple comparisons. The *p*-values for statistically significant results are in bold.

**Table 9:** Univariate logistic regression analysis of factors and miRNAs affecting endometrial cancer (N = 18).

Endometrial Cancer	OR (Odds Ratio)	Lower 95% Confidence Interval	Upper 95% Confidence Interval	*p*-Value
Age	1.09	1.01	1.17	**0.029**
First period	0.73	0.47	1.12	0.150
Last menstrual period	1.08	0.94	1.25	0.285
Number of Births	1.28	0.66	2.46	0.465
At least one birth	1.79	0.36	8.90	0.479
Number of Caesarean sections (CS)	0.58	0.18	1.86	0.362
At least one CS	0.75	0.14	3.94	0.734
At least one miscarriage	1.08	0.25	4.60	0.920
BMI	1.14	0.99	1.30	0.060
Good BMI (20–25)	0.22	0.05	1.03	0.054
Overweight (BMI => 25)	4.50	0.97	20.83	0.054
Obesity (BMI >= 30)	2.67	0.55	12.88	0.222
Hypertension	3.39	0.72	16.07	0.124
Tissue miR-21-5p (log)	0.47	0.10	2.27	0.349
Tissue miR-205-5p (log)	10.91	2.27	52.48	**0.003**
Tissue miR-222-3p	0.02	0.002	0.35	**0.006**
Serum miR-39-9p	0.39	0.03	5.94	0.495
Serum miR-21-5p	0.70	0.15	3.36	0.654
Serum miR-205-5p	1.29	0.37	4.50	0.691
Serum miR-222-3p	0.12	0.02	0.74	**0.022**

Age was only important clinical factors associated with the development of EC. Among the miRNAs examined in tissue, two showed a significant association with endometrial cancer: tissue miR-205-5p and tissue miR-222-3p. Among the serum miRNAs examined, only serum miR-222-3p showed a significant association with endometrial cancer. The *p*-values for statistically significant results are in bold.

**Table 10:** Correlation between serum and tissue miRNA concentration—Spearman correlation test.

miRNA	N	R Coefficient	*p*-Value
miR-21-5p	37	−0.23	0.176
miR-205-5p	37	0.02	0.896
miR-222-3p	37	−0.02	0.885

The table shows the correlation between miRNA concentration in serum and tissue using Spearman test. Statistical significance was not achieved.

**Table 11:** Multivariate logistic regression analysis of miRNAs associated with endometrial cancer (N = 18).

Endometrial Cancer	OR (Odds Ratio)	Lower 95% Confidence Interval	Upper 95% Confidence Interval	*p*-Value
Age	1.08	0.94	1.24	0.301
Tissue miR-205-5p	24.3	0.5	1170.34	0.107
Tissue miR-222-3p	0.002	0.000001	3.07	0.095
Serum miR-222-3p	0.002	0.000000	18.91	0.179

The obtained results showed no statistical significance.

**Table 12:** Evaluation of differences in concentrations in FIGO < 2 and FIGO => 2 groups in patients with endometrial cancer.

	Figo < 2 (N = 14)		Figo = >2 (N = 4)		*p*-Value	Benjamini–Hochberg Adjusted *p* Value ^3^
	Mean	SD	Mean	SD		
Serum miR-39-3p ^1^	4.11	0.23	4.24	0.09	0.222	0.7
Serum miR-21-5p ^2^	4.62	0.53	4.25	0.23	0.203	0.444
Tissue miR-21-5p ^2^	5.73	0.34	6	0.36	0.189	0.444
Tissue miR-205-5p ^2^	3.94	0.68	3.87	0.9	0.857	0.857
Tissue miR-222-3p ^2^	4.64	0.38	4.6	0.33	0.843	0.857
Serum miR-205-5p ^2^	2.54	0.62	1.68	0.32	**0.017**	0.17
Serum miR-222-3p ^2^	3.05	0.6	2.82	0.2	0.464	0.7

Assessment of differences in miRNA concentrations in two groups depending on FIGO < 2 and FIGO => 2. Serum miR-205-5p was initially statistically significant, but lost its significance after the Benjamini–Hochberg correction. ^1^ U Mann–Whitney test; ^2^ T-test; ^3^ Benjamini–Hochberg method. The *p*-value for a statistically significant result is in bold.

**Table 13:** Evaluation of concentration differences in G3 and non-G3 groups in patients with endometrial cancer.

	Non G3 (N = 12)		G3 (N = 6)		*p*-Value	Benjamini–Hochberg Adjusted *p* Value ^3^
	Mean	SD	Mean	SD		
Serum miR-39-3p ^1^	4.12	0.25	4.19	0.42	0.543	0.776
Tissue miR-21-5p ^2^	5.78	0.36	5.80	0.37	0.894	0.99
Tissue miR-205-5p ^2^	3.82	0.78	4.13	0.53	0.389	0.776
Tissue miR-222-3p ^2^	4.71	0.38	4.46	0.24	0.152	0.507
Serum miR-21-5p ^2^	4.53	0.55	4.55	0.42	0.935	0.99
Serum miR-205-5p ^2^	2.59	0.66	1.86	0.38	**0.024**	0.12
Serum miR-222-3p ^2^	2.93	0.56	3.15	0.5	0.441	0.776

Evaluation of different concentrations in G3 and non-G3 groups. Serum miR-205-5p concentration was initially statistically significant, but the result lost significance after Benjamin Hochberg correction. ^1^ U Mann–Whitney test; ^2^ T-test; ^3^ The Benjamini–Hochberg correction was used to account for multiple comparisons. The *p*-value for a statistically significant result is in bold.

## Data Availability

The data presented in this study are available upon request from the corresponding authors.
